# Supplementary material for: The causal role of circulating inflammatory markers in osteoporosis: a bidirectional Mendelian randomized study
Source: Front Immunol. 2024 Jul 18;15:1412298. doi: 10.3389/fimmu.2024.1412298 (PMC11291241; doi:10.3389/fimmu.2024.1412298)
Supplement: Supplementary file 1 [file DataSheet_1.zip › supplementary material/OSTEOPOROSIS-CIM/GCST90274846.tsv.gz_leave_one_out.pdf]

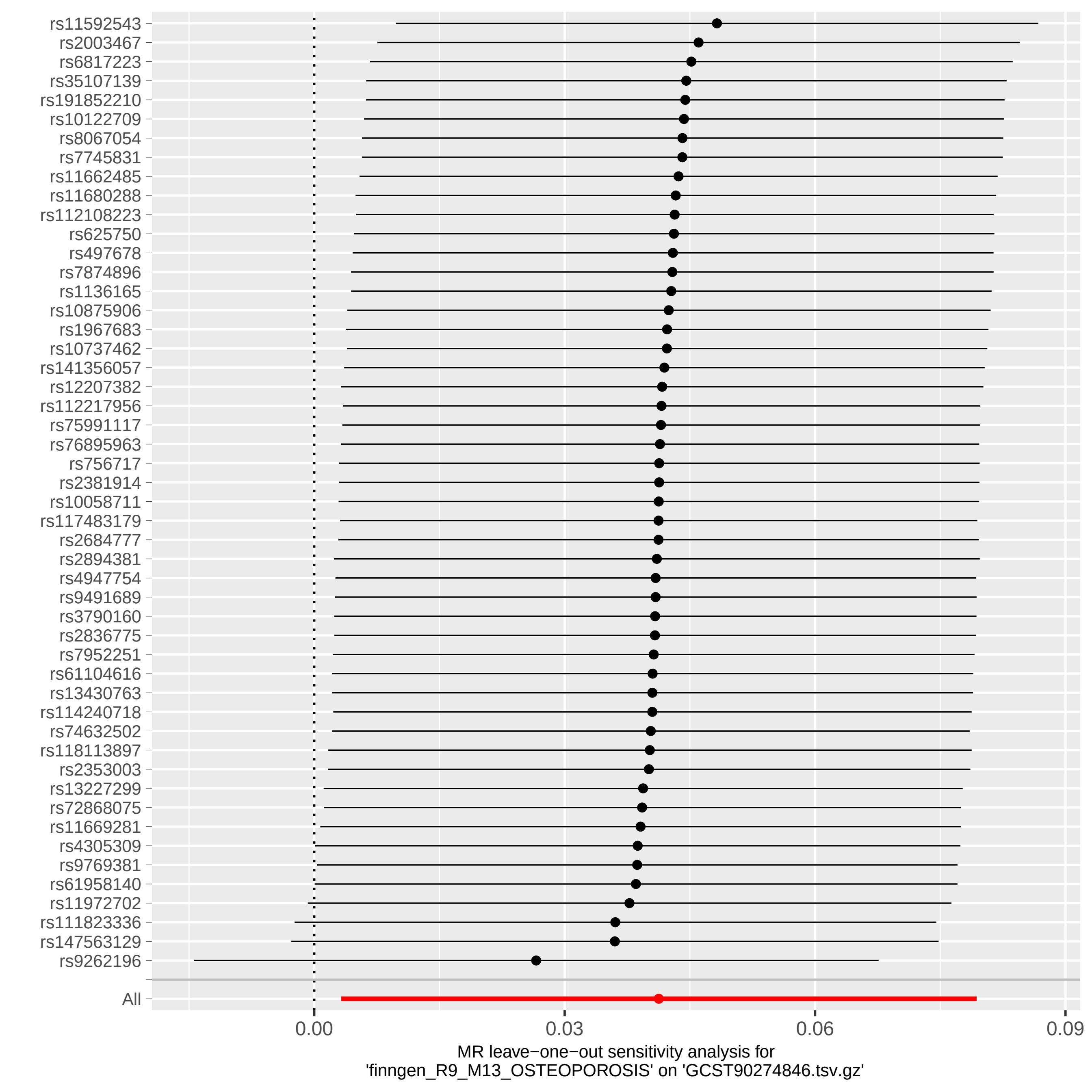
rs11592543
rs2003467
rs6817223
rs35107139
rs191852210
rs10122709
rs8067054
rs7745831
rs11662485
rs11680288
rs112108223
rs625750
rs497678
rs7874896
rs1136165
rs10875906
rs1967683
rs10737462
rs141356057
rs12207382
rs112217956
rs75991117
rs76895963
rs756717
rs2381914
rs10058711
rs117483179
rs2684777
rs2894381
rs4947754
rs9491689
rs3790160
rs2836775
rs7952251
rs61104616
rs13430763
rs114240718
rs74632502
rs118113897
rs2353003
rs13227299
rs72868075
rs11669281
rs4305309
rs9769381
rs61958140
rs11972702
rs111823336
rs147563129
rs9262196
All
0.00
0.03
0.06
0.09
MR leave−one−out sensitivity analysis for
'finngen_R9_M13_OSTEOPOROSIS' on 'GCST90274846.tsv.gz'
